# Spatial-frequency features of radiation produced by a step-wise tapered undulator

**DOI:** 10.1107/S1600577521001958

**Published:** 2021-03-26

**Authors:** Andrei Trebushinin, Svitozar Serkez, Mykola Veremchuk, Yakov Rakshun, Gianluca Geloni

**Affiliations:** a Budker Institute of Nuclear Physics, Novosibirsk, Russia; b Novosibirsk State University, Novosibirsk, Russia; c European XFEL, Hamburg, Germany; d Taras Shevchenko National University of Kyiv, Kyiv, Ukraine

**Keywords:** tapered undulator, superconducting undulator, wavefront propagation

## Abstract

A scheme to generate wide-bandwidth radiation using a step-wise tapered undulator with a segmented structure is proposed. This magnetic field configuration allows to broaden the undulator harmonic spectrum by two orders of magnitude, providing 1 keV bandwidth with spectral flux density exceeding 10^16^ photons s^−1^ mm^−2^ (0.1% bandwidth)^−1^ at 5 keV on the sample. Such a magnetic setup is applicable to superconducting devices where magnetic tapering cannot be arranged mechanically. The resulting radiation with broadband spectrum and flat-top shape may be exploited at a multipurpose beamline for scanning over the spectrum at time scales of 10–100 ms. The radiation from a segmented undulator is described analytically and derivations with numerical simulations are verified. In addition, a start-to-end simulation of an optical beamline is performed and issues related to the longitudinally distributed radiation source and its image upon focusing on the sample are addressed.

## Abstract 2

Spatial frequency radiation properties from a step-wise magnetic structure of an undulator are studied. This magnetic scheme is the most suitable for the superconducting undulators and provides the radiation properties that facilitate developing future beamlines dedicated to micro-probing.

## 1. Introduction   

Synchrotron radiation (SR) serves as a powerful tool for investigating materials with X-rays. High photon flux and photon energy tuning capabilities provide these sources uncontested benefits over laboratory X-ray sources. SR sources facilitate spectroscopy techniques such as X-ray absorption spectroscopy (XAS), X-ray absorption near-edge structure (XANES), X-ray emission spectroscopy (XES) as well as X-ray diffraction techniques. Moreover, fast monochromators allow to speed up data acquisition, for example for extended X-ray absorption fine structure (EXAFS) spectroscopy resulting in quick-EXAFS (Frahm, 1988 ▸). This requires fast scanning over 1 keV bandwidth at a sub-second time scale with a sufficient photon flux on the sample. In this work, we propose an advanced superconducting undulator scheme for the experiments where fast scanning over the spectrum along with micrometre-scale focusing is required. We suggest that this source may be installed for future beamlines specialized in micro- and nano-probing, *e.g.* I18 at DIAMOND (Mosselmans *et al.*, 2009 ▸), P06 and P11 at PETRA III (Schroer *et al.*, 2010 ▸; Burkhardt *et al.*, 2016 ▸) and ID13 at ESRF (Flot *et al.*, 2010 ▸).

The number of periods in an undulator defines a resonance bandwidth for the *n*th harmonic, expressed as 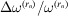 ≃ 1/*nN*, where 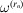 denotes a resonant frequency and *N* stands for the number of periods. In absolute units, this bandwidth is only of the order of 10 eV for an undulator with 100 periods at the resonance of 1000 eV. One way to perform scanning experiments is to set the magnetic field of the undulator in a tracking mode and scan it together with a monochromator. Scanning, however, raises a challenging technical issue connected with on-the-fly magnetic field tuning, especially at sub-second scales. Another way consists of obtaining the radiation with a bandwidth that covers the desired scan range – about 1 keV – and performing the scan with a fast monochromator. The SR community already exploits undulator tapering around a given harmonic in order to provide the required bandwidth for the experiments. In the tapering mode the magnetic field gradually changes along an electron trajectory in the range from *B*_min_ to *B*_max_. The resonance effectively widens satisfying the resonance conditions for all values of the magnetic field range. The tapering mode for undulators has been studied and used at SR sources (Walker, 1988 ▸; Bosco & Colson, 1983 ▸; Boyanov *et al.*, 1994 ▸).

Here we propose to generate a magnetic field with a step-wise magnetic profile employing a segmented undulator. The radiation from the step-wise tapered magnetic field creates a series of consecutive resonances, summing up to a broad spectrum. Depending on the number of segments, this setup may provide 1 keV spectral bandwidth. The step-wise tapered technique is particularly suitable for setups utilizing superconducting undulators, where the undulator gap is fixed and the only way to create the field gradient is to change the value of the current in the superconducting coils.

In this work, we present our calculations with design parameters for a superconducting undulator project for the SR source SKIF (the Russian acronym for Siberian Circular Photon Source) in Novosibirsk. We design this superconducting undulator in collaboration with the undulator group in Budker Institute of Nuclear Physics (Shkaruba *et al.*, 2018 ▸; Ivanyushenkov *et al.*, 2015 ▸; Mezentsev & Perevedentsev, 2005 ▸).

## 2. Concept   

**Figure 1:**
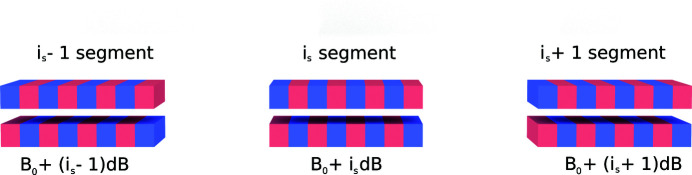
Scheme of a step-wise tapered undulator.

We propose to divide an undulator with constant period length λ_*w*_ into *n*_s_ segments with *N*_s_ periods each. Thus, we create a multi-segment structure as depicted in Fig. 1 ▸. With this we aim to obtain a sequence of overlapping resonances, resulting in a flat-top spectrum with minimum on-top fluctuation.

Each segment of the superconducting undulator will be equipped with a separate power supply or correction coils (or auxiliary coils) to control the value of the magnetic field. The magnetic field varies as 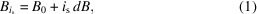
where *i*_s_ = 0,…, *n*_s_ − 1 is the segment number, *B*_0_ and *dB* are, respectively, the field in the first segment and the field leap to the next segment in sequence. We propose to use two windings: a main one with the reference field *B*_0_ and an auxiliary one which shapes this step-wise magnetic structure with *dB* increments. Overall, the auxiliary coil in the last segment should provide the additional field *n*_s_ *dB* which is less than 20% of the main field for the third harmonic at the energy around 4.5 eV. The magnetic field sets the corresponding value of current in the auxiliary coils. Dividing the undulator into two windings seems to be more efficient in the sense of thermal stability of a cryostat and in terms of cost when one needs to reduce the number of high-current power suppliers.^1^

Each segment has a resonance at the *n*th harmonic with relative bandwidth 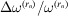 ≃ 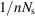 resulting in 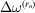 ≃ 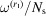, where 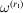 and 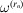 are the resonance frequencies for the first harmonic and the *n*th harmonic. We found that to effectively generate a wide-bandwidth flat-top spectrum the individual resonances of each segment should be shifted by their full width at half-maximum (FWHM). We justify this criterion in Appendix *B*. Hence, the resulting combined undulator bandwidth for all odd harmonics becomes 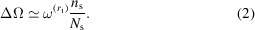
It is worth noting that this undulator can also be used in uniform mode (*dB* = 0). So, this device is multi-functional.

## 3. Analytical description of the segmented undulator   

In our derivation, we exploit the resonance approximation. This approximation requires a large number of periods (*N*_s_ ≫ 1). We also use a filament electron beam approximation in our analytical derivation. In other words, the emittance of the electron beam is much smaller than the ‘emittance’ of radiation (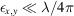) and we neglect energy spread effects. These assumptions will be justified later on by comparison with numerical calculations. We present a list of the undulator parameters in Table 1 ▸.

We denote the total length and the period of the insertion device as *L* and λ_*w*_, respectively. For the sake of clarity, we provide SKIF electron beam parameters in Table 2 ▸.

*E* and *dE* denote electron beam energy and its spread, respectively, while β_*x*_ and β_*y*_ are the (almost constant) horizontal and vertical beta functions in the straight section, and ε_*n*_ and κ refer to the natural emittance and the coupling factor, respectively.

### 3.1. Segmented undulator spectrum: theoretical model and its verification with a numerical simulation   

#### 3.1.1. Analytically calculated on-axis spectrum   

**Figure 2:**
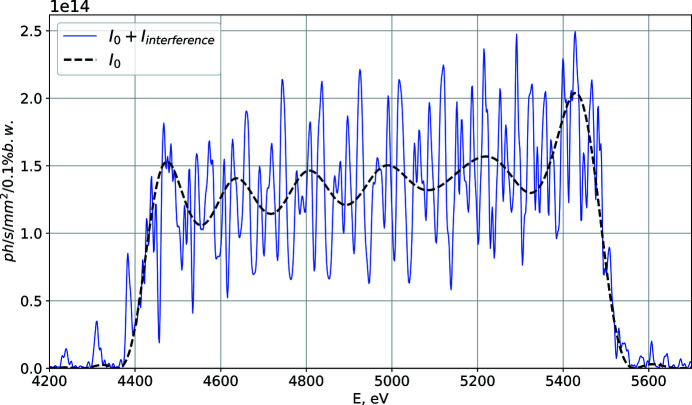
On-axis spectrum of the third harmonic within our theoretical model. The black dashed line shows the shape of the spectrum in the case when the contribution of the interference term is neglected. The blue solid line shows the spectrum with the effects of interference included.

We provide all derivations in a Gaussian unit system. The derivation of undulator radiation in the frequency domain may be found in textbooks on SR although we follow the approach and notation of Geloni *et al.* (2007 ▸). We derive the radiation distribution emitted by a single electron on-axis from a segmented undulator as a sum of the fields from *n*_s_ separate segments the centres of which are located at the distance *z*_0_(*i*_s_) from an observer, 
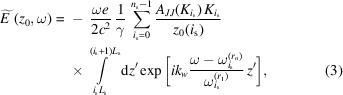
where γ is the Lorentz factor, *e* is the electron charge, *c* is the speed of light and *k*_*w*_ = 2π/λ_*w*_, 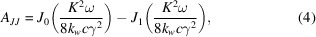
where *J*_*n*_ is a Bessel function of the first kind of order *n*. 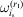 is the fundamental resonant frequency of the *i*_s_th segment, *L*_s_ = *N*_s_λ_*w*_. 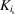 is the undulator parameter of the *i*_s_ segment. 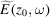 is the slowly varying envelope of the Fourier transform of the electric field. Here we consider a fixed polarization component. We give an analytical expression for the radiation from a single segment in Appendix *A* [App appa]. By taking the integral in equation (3) we obtain 
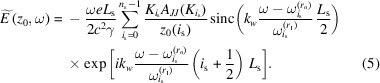
The radiation from each segment has a resonance with a relative bandwidth ∼1/*nN*_s_ and shifted from the reference resonance by 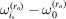. Also, the radiation from each segment has a phase shift 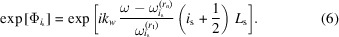
Within the filament beam model, the resulting spectral flux density in units of number of photons per second per unit surface area per unit relative bandwidth is given by 
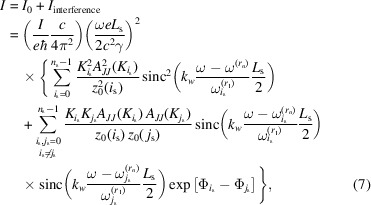
where ℏ = *h*/2π is Planck’s constant. The spectral flux density is depicted in practical units (*i.e.* in photons per second per squared millimetre per 0.1% relative bandwidth) in Fig. 2 ▸.

The blue solid line in Fig. 2 ▸ represents the effect of spectral interference of the segments, while the black dashed line shows the shape of the spectrum without the interference term in equation (7).

Our analytical model is relatively rough as we exploited the filament beam approximation and the resonance approximation, assuming a large number of undulator periods, while here we only consider 18 periods per segments. Nevertheless, this approach shows the main effect of spectral interference that could impede implementing this scheme. In the following subsections, we provide a more thorough investigation of the step-wise tapered magnetic field configuration using a numerical simulations approach.

#### 3.1.2. Numerical simulations of the on-axis spectrum   

We performed our simulation using a multi-physics software for numerical calculation of electron beam dynamics, free-electron lasers and SR sources performance – *OCELOT* (Agapov *et al.*, 2014 ▸, https://github.com/ocelot-collab). The *OCELOT* software allows to calculate SR from a filament beam in an arbitrary magnetic field and to propagate this radiation through an optical beamline.

**Figure 3:**
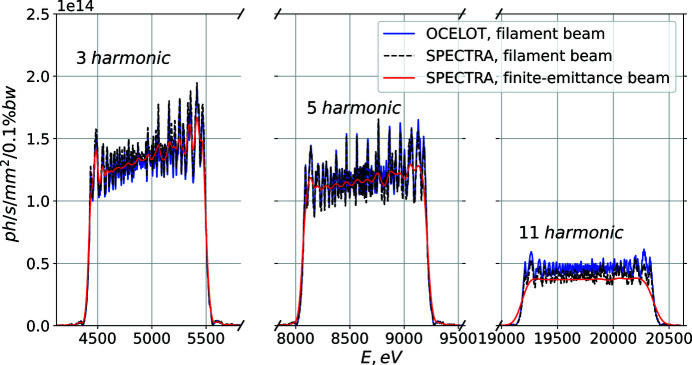
Simulation results of the on-axis spectrum for third, fifth and eleventh harmonics. We assume that the segmented undulator is gapless, *i.e.* the distance between the cells is negligible, hence no additional phase shift is present.

**Figure 4:**
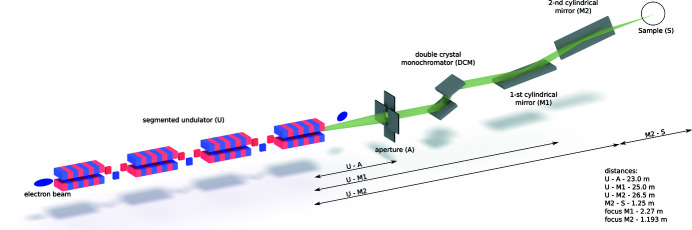
Illustration of the beamline optics. From left to right: step-wise tapered undulator, aperture at 23 m from the end of the undulator, first cylindrical mirror – 2 m from the aperture, second cylindrical mirror – 1.5 m from the first mirror, distance from the second mirror to the sample 1.25 m.

We provide simulation results for the on-axis spectrum emitted in the segmented undulator, Fig. 3 ▸, with the device parameters listed in Table 1 ▸. We consider a range of energies from the Ti *K*-edge, 4.5 keV, up to the edges of heavier elements, 20 keV. As we showed in equation (2), it is possible to obtain 1 keV spectral bandwidth for all desired photon energies. The values of *dB*/*B*_0_ for these simulations are listed in Table 3 ▸. We crosschecked our *OCELOT* results with *SPECTRA* (Tanaka & Kitamura, 2001 ▸) that are depicted in Fig. 4 ▸. The red line shows the effect of the finite electron beam emittance and energy spread (SKIF electron beam parameters). One can see that assuming an electron beam of finite size results in a less modulated spectral shape without notable flux loss. Given that the modulated spectrum is undesired for users, we use the filament beam as a conservative approximation.

Though the final design of this undulator does not contain free space gaps or phase shifters between the segments (V. Shkaruba & N. Mezentzev, private communication), we provide further analysis of the spectral shape including the influence of free space gaps in Appendix *B* [App appb].

## 4. Wavefront propagation simulation: focusing   

Beamlines for the energy range between 5 keV and 40 keV typically consist of an entrance aperture, a double-crystal monochromator and a focusing system, *e.g.* a pair of Kirk­patrick–Baez mirrors. We present a simplified scheme of the SKIF beamline in Fig. 4 ▸. In this section we study imaging of the undulator radiation on the sample using the spontaneous emission and radiation propagation modules of *OCELOT*. In particular, we model the third harmonic radiation of an intersection-less segmented undulator with optimized magnetic field increments. We consequently propagate the emitted radiation through the beamline up to the sample location. We will not account for the monochromator in our simulations as it does not affect the image formation process.

As discussed above, in our simulations we use the filament beam model; partially because calculations of radiation by a finite electron beam (Chubar *et al.*, 2011 ▸) are computationally expensive, and partially by aspiration to show features related to the insertion device itself, on which we focus in this contribution. One can estimate the radiation beam size and divergence for a given electron beam on each optical element using ray-tracing codes, for example *SHADOW* (Sanchez del Rio *et al.*, 2011 ▸).

### 4.1. Radiation on the sample in the case when no focusing is applied   

**Figure 5:**
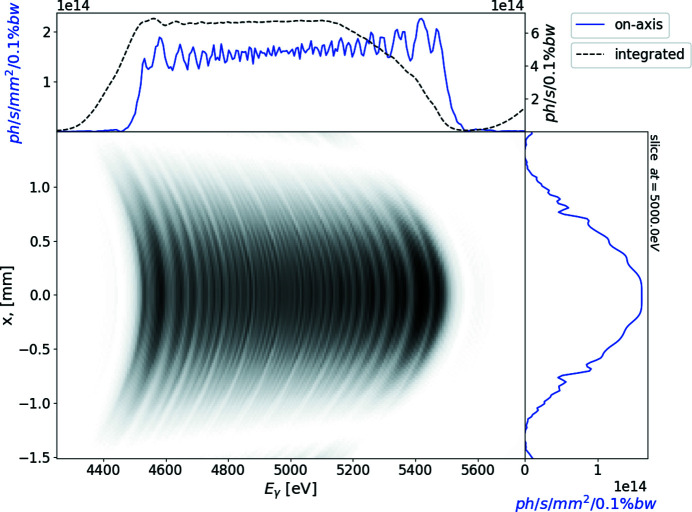
Single electron intensity distribution from the segmented undulator at 25 m from the exit of the undulator as a function of photon energy and transverse coordinate. The top subplot represents on-axis spectral flux density (blue line) and the spectrum transversely integrated over a simulation window – brightness (black line); the right-hand subplot depicts the field distribution at 5000 keV (blue line).

In the case of no focusing, the transverse size of the radiation on the sample is about 1 mm FWHM after 25 m of free-space propagation. In this case the calculated spectral flux density is of the order of 2 × 10^14^ photons s^−1^ mm^−2^ (0.1% bandwidth)^−1^. Owing to the angular dependence of the undulator resonance condition, the spectral flux density integrated over both transverse directions exhibits minor modulations, shown in Fig. 5 ▸.

### 4.2. The field distribution on the sample after focusing   

**Figure 6:**
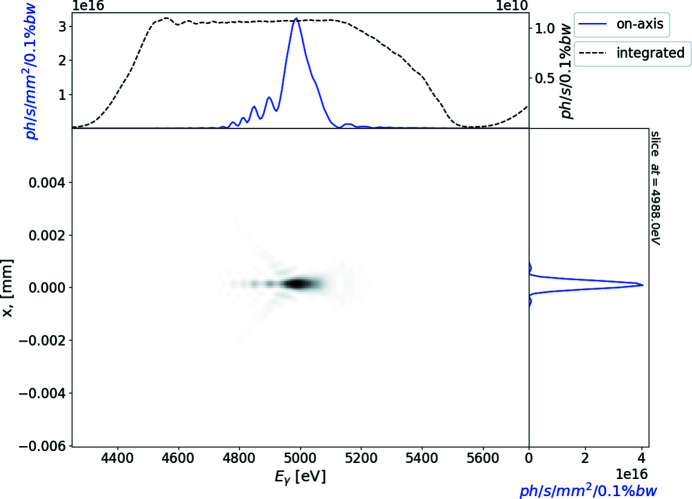
Simulation of the radiation from the segmented undulator after focusing when there is no aperture in the far zone. Axis convention is the same as for Fig. 5 ▸.

In order to increase the spectral flux density, one may introduce focusing elements to concentrate the radiation on a smaller spot on the sample. When we form an image in the focal plane, we reconstruct a demagnified version of the object plane. However, the segmented undulator is a non-uniform source and has chromatic aberrations, which we discuss in Appendix *C* [App appc]. In fact, the longitudinal source scale in the image is reduced by the demagnification factor 1/*D* ^2^ = *b* ^2^/*a* ^2^, where *a* is the distance from the source to the centre of the optical system and *b* is the distance from the centre of the optical system to the sample. The longitudinal offset is then in the order of centimetres. In Fig. 6 ▸ we plot the space–frequency distribution of the radiation on the sample. The on-axis photon flux is extremely dispersed across the energy range and the spot position varies longitudinally.

**Figure 7:**
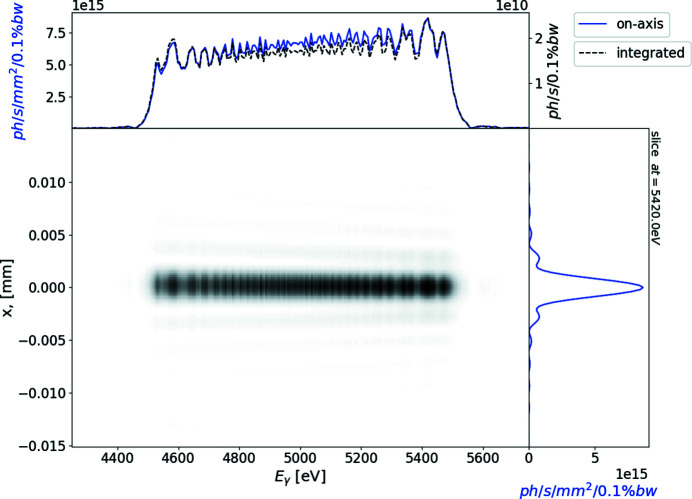
The radiation after focusing in the case of an aperture of 0.3 mm × 0.3 mm placed at 23 m from the exit of the undulator. The sample is located in the focus (1.193 m from the second mirror). It is fair to say we are looking near the *k*_⊥_ = 0 components – spatial filtering. Thus, we see two effects on the sample: spatial filtering and diffraction on the aperture. Axis convention is the same as for Fig. 5 ▸ except the blue line on the right-hand subplot which is plotted for the slice with maximum intensity.

As we already noted, the segmented undulator is a longitudinally distributed source, therefore the radiation from each segment converges at different distances from the focusing element. To mitigate this effect we introduce an aperture with size 0.3 mm × 0.3 mm in the far zone of the undulator. The aperture increases the Rayleigh length of the image waist as well as the imaginary source, effectively reducing chromatic abberration, see Fig. 14 in Appendix *C* [App appc]. Thus, the full spectrum of the radiation is uniformly concentrated in one transverse spot, Fig. 7 ▸.

**Figure 8:**
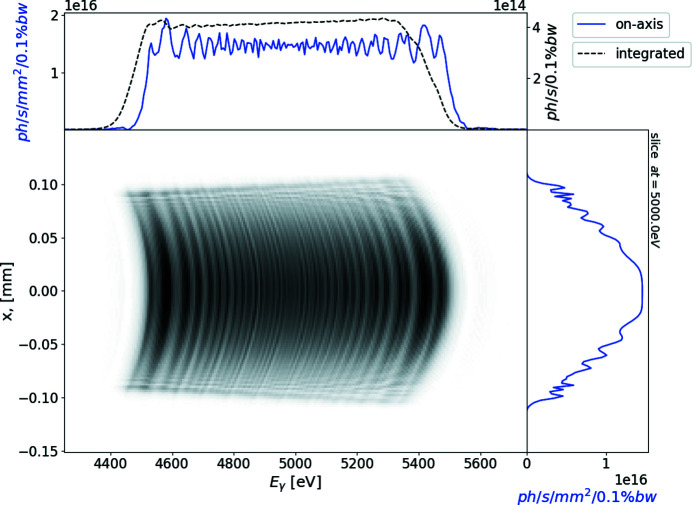
Simulation of the radiation from the segmented undulator after collimation. An aperture of 2 mm × 2 mm is placed at 23 m from the exit of the undulator. The sample is located out of focus (1.1 m from the second mirror). Axis convention is the same as for Fig. 5 ▸.

The notable disadvantage of reducing the aperture size is a significant photon flux loss (over 98%). Therefore, we propose to place the sample slightly out of focus after a much milder collimation with a wider aperture. In this case, we enhance the photon flux density converging the radiation on a smaller spot but we do not significantly lose photons on the aperture to reduce chromatic aberration. This is because we do not re-image the source and gain the benefits of spatial-frequency properties of radiation in the far zone. We present simulation results of the out-of-focus image on Fig. 8 ▸ where we applied an aperture with a size of 2 mm × 2 mm (only 36% photon flux loss). In this simulation, we change the longitudinal position of the sample and place it at 1.1 m from the second mirror although, on the actual beamline, the same effect can be reached by changing the angle of incidence on the mirror.

**Figure 9:**
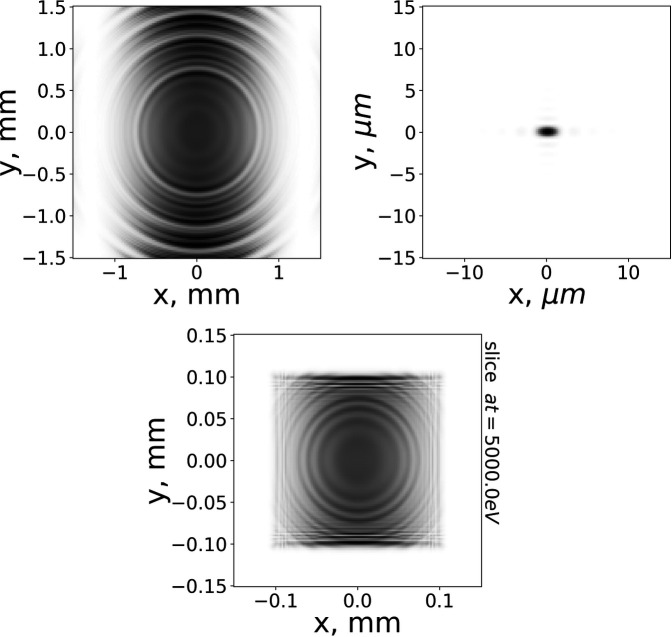
Transverse intensity profiles of monochromatic radiation at 5000 eV corresponding to Figs. 5 ▸, 7 ▸ and 8 ▸ from left to right, top to bottom, respectively.

Additionally, we present the transverse intensity distributions for the calculated fields in Fig. 9 ▸.

## 5. Discussion   

**Figure 10:**
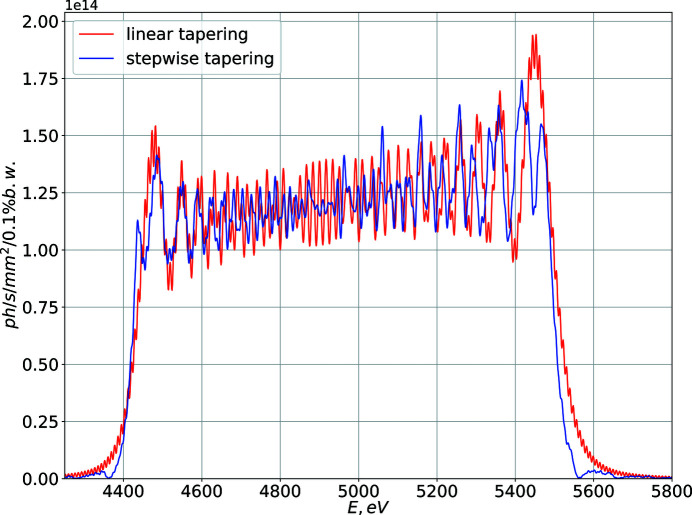
Comparison of an on-axis undulator spectrum in the case of a linear (red line) and step-wise (blue line) undulator tapering for filament electron beam approximation. Aside from different tapering profiles, the simulation parameters are identical to those used to generate, for example, Fig. 3 ▸.

**Figure 14:**
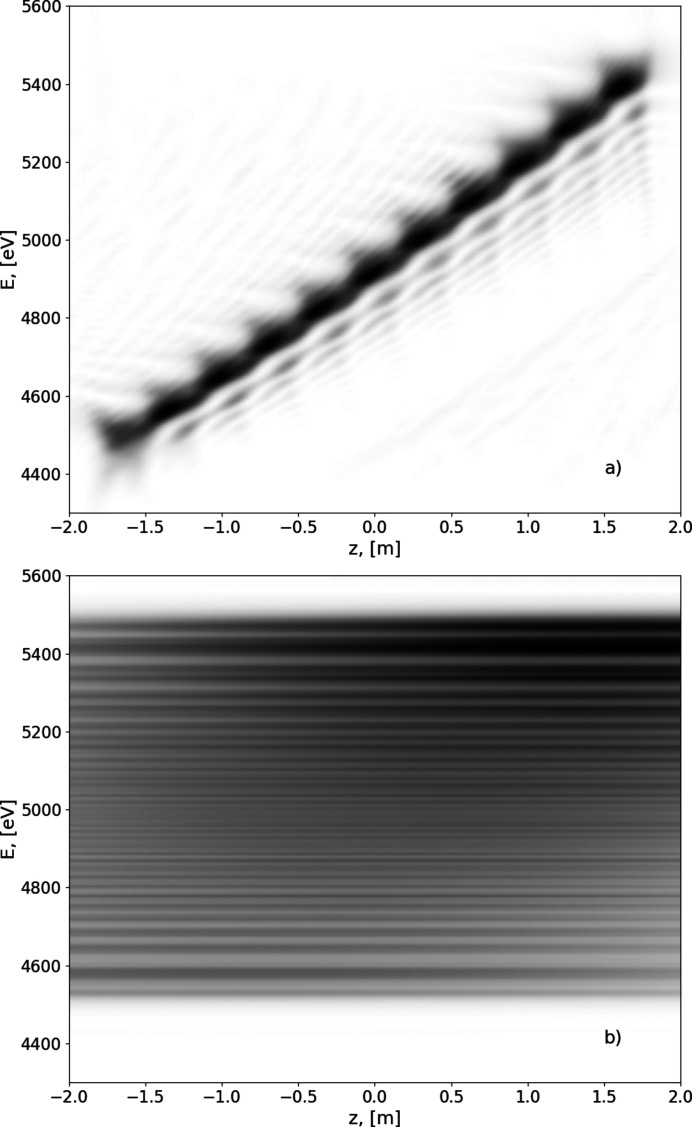
On-axis radiation intensity distribution depending on energy of radiation (vertical axes) and the longitudinal position in the undulator (horizontal axes) (*a*) without the aperture, (*b*) with the aperture.

Undulator tapering is a known method to broaden the spectrum of the undulator radiation (Nonaka *et al.*, 2016 ▸; Caliebe *et al.*, 2019 ▸) and it is carried out as a gradual incremental change of the *K* parameter over the undulator length. While we propose to implement a qualitatively different step-wise tapering, the resulting performance is very similar compared with that of the linear tapering, as illustrated in Fig. 10 ▸

Step-wise tapering, being easier to implement using an electromagnetic undulator, yields comparable amplitude of spectrum modulation. As already discussed above (see Fig. 3 ▸), the latter is further reduced due to the finite electron beam emittance.

## 6. Conclusion   

In this contribution, we proposed a scheme for generating broadband undulator radiation and delivering it to the sample. The scheme relies on a segmented superconducting undulator where the undulator segments are detuned by the FWHM of their individual spectral bandwidth with respect to each other. This segmented structure emits partially overlapping narrow-bandwidth fragments of the final spectrum. The resulting on-axis spectrum has a broadband modulated on-axis distribution, while transversely integrated radiation from a finite electron beam exhibits a smooth flat-top spectrum shape. The spectral density is modulated due to interference between the segments. If undulator segments are spatially separated, they should be accompanied by phase-shifters. Numerical simulations indicate the trade-off between the photon density on the sample and the flatness of the resulting broadband spectrum due to both modulated spectrum and a longitudinally distributed photon source. For the undulator with 11 segments and 18 periods in each segment, we obtained 1 keV spectral bandwidth with photon flux exceeding 2 × 10^14^ photons s^−1^ (0.1% bandwidth)^−1^ for filament electron beam calculations. This contribution serves as a conceptual design for the beamline dedicated to micro-probe experiments at SKIF.

## Figures and Tables

**Figure 11:**
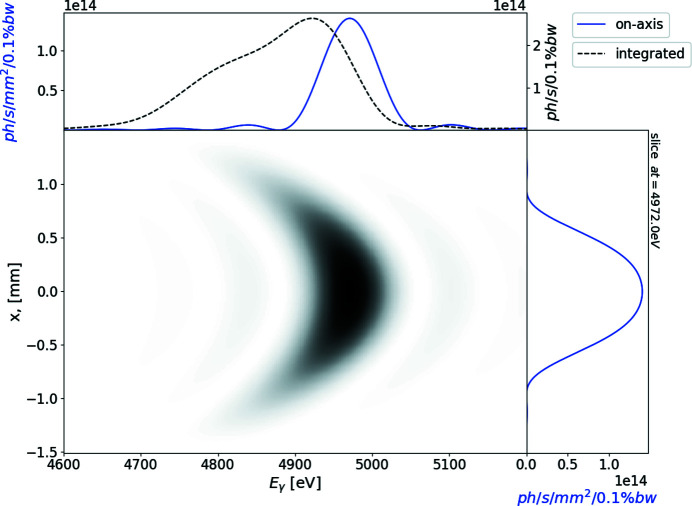
Single electron field distribution depending on photon energy (horizontal axis) with respect to the transverse coordinate *x* for *y* = 0 (vertical axis). Top and right subplots depict the spectrum and transverse intensity distribution at undulator resonance at its maximum intensity, respectively. In the top subplot, the blue line is an on-axis spectrum and the black line is the integrated over simulation window intensity.

**Figure 12:**
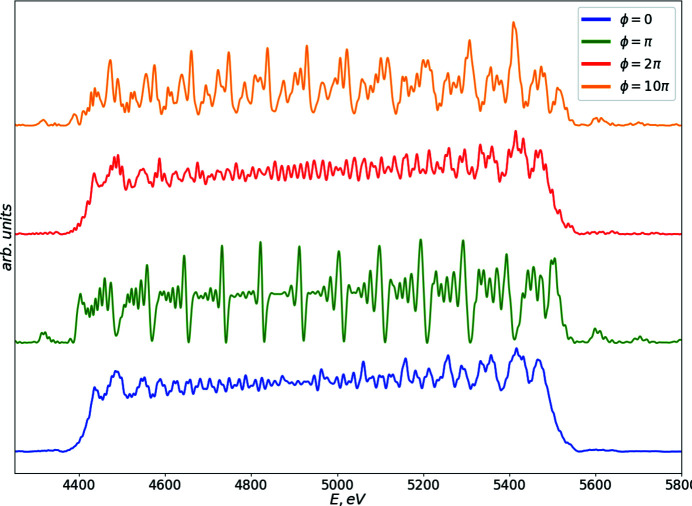
Simulation results of the on-axis spectrum from the segmented undulator including drift segments. The phase subscription denotes the phase advance due to drift segments.

**Figure 13:**
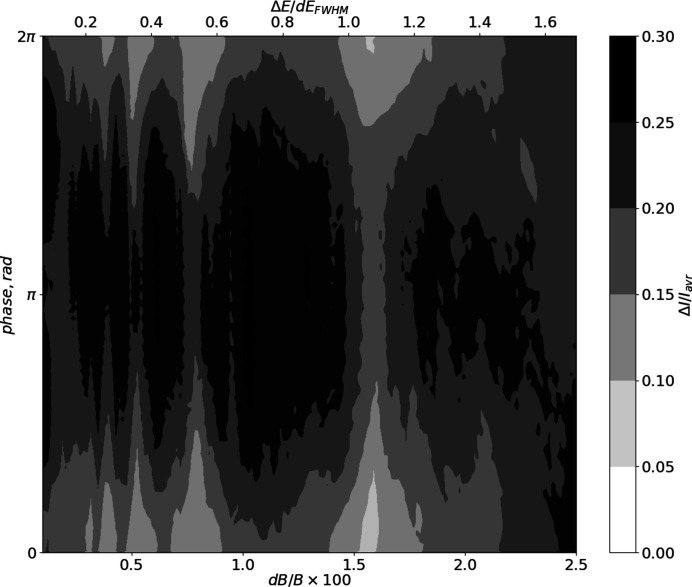
The colour representation of the spectrum modulation amplitude (standard deviation of the spectrum plateau normalized on the average intensity) as a function of segment field detuning (*x*-axis, bottom scale), single segment spectrum detuning (*x*-axis, top scale) and the radiation phase shift between the segments (*y*-axis)

**Table 1:** Undulator parameters

*B*_0_ (T)	*dB*/*B*_0_	λ_*w*_ (mm)	*L* (m)	*N*_s_	*n*_s_
1.05	0.0158	18	3.56	18	11

**Table 2:** SKIF parameters

*E* (GeV)	δ*E*/*E*	β_*x*_ (m)	β_*y*_ (m)	ε_*n*_ (pm rad)	κ	*I* (A)
3	1.35 × 10^−3^	15.66	2.29	105.45	0.1	0.4

**Table 3:** Harmonic detuning

Harmonic	3	5	11
*dB*/*B*_0_ × 100%	1.58	0.95	0.42

## Footnotes

1 This scheme with auxiliary coil initially is proposed by N. Mezentsev and V. Shkaruba and will be discussed in more technical details in future papers.

## Appendix A Radiation of a single electron in a separate segment

The electric field emitted by a single electron moving in a planar undulator is linearly polarized within the resonance approximation, and its magnitude can be calculated as an integral over the particle trajectory in the undulator (for example, Onuki & Elleaume, 2003 ▸),

where  = , γ is the Lorentz factor, *e* is the electron charge, *c* is the speed of light and *k*_*w*_ = 2π/λ_*w*_,

with *J*_*n*_ the Bessel function of the first kind of order *n*. The field is calculated at an observation point *z*_0_, **θ** = (*x*/*z*_0_, *y*/*z*_0_), *z*_0_ is measured from the undulator centre, located at *z* = 0. The resonance frequency is denoted as ω. The polarization lies in the **e**_*x*_ plane. The notation of the field representation and the coordinate system used in our derivation coincides with one established by Geloni *et al.* (2007 ▸). Taking the integral in equation (8) we obtain the usual expression for the field distribution from the single electron in the far zone,

We illustrate the corresponding intensity distribution as a function of photon energy and of the transverse coordinate *x* while *y* = 0, Fig. 11 ▸.

## Appendix B Phase advance in the drift between the segments

Heretofore, both analytical Fig. 2 ▸ and numerical Fig. 3 ▸ results assumed zero drift distance between the segments. However, if the undulator segments are separated by intersections of length *L*_*d*_, an additional phase term appears in the field expression equation (5) (on-axis),

where  =  is the first harmonic resonant wavelength of the upstream undulator cell. The segments interfere and, if destructively phased, introduce fluctuations in the spectrum. In Fig. 12 ▸ we show the influence of the phase shifts between radiation pulses emitted in adjacent segments on the spectral shape.

One can compensate these phase shifts by installing phase shifters in the intersections. Thus, each phase shifter provides a 2*n*π phase advance, where *n* is an integer number, for every photon energy the undulator operates. However, the undulator without the drifts and phase shifters constitutes a more cost-effective solution (V. Shkaruba & N. Mezentzev, private communication).

In Fig. 13 ▸ we show the dependence of spectrum modulation standard deviation on two undulator parameters: radiation phase advance between undulator segments in radians and the relative difference between the magnetic field of two neighbouring undulator segments. The spectrum modulation is minimized by shifting radiation spectra of adjacent cells by approximately one FWHM of their bandwidth and by introducing a phase advance between segments as the smallest multiple of 2π. This corresponds well with our assumptions made in Section 2.

## Appendix C Radiation at the imaginary source

It is convenient to study the radiation properties on the sample by examining those at the imaginary radiation source. Once we have calculated the radiation field in the far zone we propagate it back to the undulator centre by means of the free space propagator (Voelz, 2011 ▸; Schmidt & Daniel, 2010 ▸). The resulting field we call the field distribution at the imaginary source. The shape of the field distribution at the imaginary source facilitates interpretation and understanding of the frequency and spatial structure of the radiation in the image plane after focusing. The field at the imaginary source consists of the contributions from all the segments, with their minimum waists located in the centres of these segments. Since the central frequency of each contribution also depends on the segment, we deal with a chromatic aberration. To illustrate this we depict the on-axis field distribution at the different locations within the undulator (*i.e.* the imaginary source) with respect to the photon energies, Fig. 14 ▸.

Upon imaging, similar dependence would be present at the sample location. To mitigate this effect we introduce an aperture in the far zone. The aperture, 0.3 mm × 0.3 mm, at 25 m from the source smooths the on-axis spectrum at the imaginary source, Fig. 14 ▸(*b*). In this way, we effectively increase the Rayleigh length of the imaginary source.
